# Concentration-Dependent Type 1 Interferon-Induced Regulation of MX1 and FABP3 in Bovine Endometrial Explants

**DOI:** 10.3390/ani11020262

**Published:** 2021-01-21

**Authors:** Simone Tamara Schabmeyer, Anna Maria Kneidl, Julia Katharina Schneider, Sandra Kirsch, Yury Zablotski, Wolfram Petzl, Frank Weber, Holm Zerbe, Marie Margarete Meyerholz

**Affiliations:** Clinic for Ruminants with Ambulatory and Herd Health Services, Centre for Clinical Veterinary Medicine Ludwig Maximilians University Munich, 85764 Oberschleissheim, Germany; Simone.Schabmeyer@campus.lmu.de (S.T.S.); A.Kneidl@med.vetmed.uni-muenchen.de (A.M.K.); Schneider.Julia@campus.lmu.de (J.K.S.); Sandra.Kirsch@med.vetmed.uni-muenchen.de (S.K.); Y.Zablotski@med.vetmed.uni-muenchen.de (Y.Z.); wpetzl@lmu.de (W.P.); weber@lmu.de (F.W.); h.zerbe@lmu.de (H.Z.)

**Keywords:** endometrial explant model, embryo–maternal communication, type 1 interferon signaling, type 1 interferon receptor subunits 1/2 (IFNAR1/2), 3R principle, cattle, interferon τ, interferon α, interferon stimulated gene (ISG), subfertility

## Abstract

**Simple Summary:**

Low fertility rates in high-yielding cows have been reported for many years. Due to its impact on animal welfare, this topic is relevant to the dairy industry, veterinary medicine, and consumers. This study was designed to apply a highly defined model to simulate complex mechanisms that occur in the inner layer of the uterus (endometrium) during pregnancy recognition. Samples were taken from animals with healthy uteri at the slaughterhouse and challenged in vitro. The endometrial gene expression of selected target genes differed according to the differing concentrations of the challenging admixtures. The findings indicated that the bovine embryonic pregnancy signal might compete with similar infection-associated signals for binding capacity at the receptor level, which might be relevant to pregnancy outcomes. In conclusion, an endometrial explant model was successfully applied to answer questions related to fertility in dairy cattle. According to the 3R principle (replacement, reduction, refinement), further studies could lead to new diagnostic and therapeutic strategies for tackling subfertility in dairy cows without the need for animal experiments.

**Abstract:**

The inadequate maternal recognition of embryonic interferon τ (IFNτ) might explain subfertility in cattle. This study aimed at modeling the inducibility of type 1 interferon receptor subunits 1/2 (IFNAR1/2), mimicking competition between IFNτ and infection-associated interferon α (IFNα), and simulating type 1 interferon pathways in vitro. Endometrial explants (n = 728 from n = 26 healthy uteri) were collected at the abattoir, challenged with IFNτ and/or IFNα in different concentrations, and incubated for 24 h. Gene expression analysis confirmed the inducibility of IFNAR1/2 within this model, it being most prominent in IFNAR2 with 10 ng/mL IFNα (*p* = 0.001). The upregulation of interferon-induced GTP-binding protein (MX1, classical pathway) was higher in explants treated with 300 ng/mL compared to 10 ng/mL IFNτ (*p* < 0.0001), whereas the non‑classical candidate fatty acid binding protein 3 (FABP3) exhibited significant downregulation comparing 300 ng/mL to 10 ng/mL IFNτ. The comparison of explants challenged with IFNτ + IFNα indicated the competition of IFNτ and IFNα downstream of the regulatory factors. In conclusion, using this well-defined explant model, interactions between infection-associated signals and IFNτ were indicated. This model can be applied to verify these findings and to mimic and explore the embryo–maternal contact zone in more detail.

## 1. Introduction

Maintaining high fertility rates in dairy cowherds is a recurrent task for every farmer and veterinarian. Inflammatory processes of the endometrium lead to subfertility in cows, which might be due to inadequate maternal recognition of the embryonic signal, interferon τ (IFNτ). The restocking rate because of fertility problems in cattle is currently 20% [1,2]. It has been reported in multiple studies that low fertility rates in cattle are associated with classical postpartum diseases, such as mastitis [3], the retention of fetal membranes, metritis, and endometritis typically caused by bacterial infections [4,5,6,7]. In the postpartum period of 60–100 days, endometritis can still be present in a subclinical manner. As recently reviewed by Wathes and coworkers [8], not only bacterial but also viral infections can impact fertility parameters. Bovine herpes virus type‑4 (BHV‑4) and bovine viral diarrhea virus (BVDV) have been associated with metritis and endometritis [9,10]. Schmallenberg virus (SBV) and bluetongue virus (BTV) have been described to impact early embryonic survival [11,12]. However, the mechanisms linking viral infections and bovine subfertility remain to be investigated. It has been further reported that fertilization rates in dairy cows are generally high, but the percentage of early pregnancy loss before implantation is ~40% [13,14,15]. This could indicate the malfunctioning of embryo–maternal crosstalk. Adequate maternal recognition, the transmission of early embryonic signals, and the establishment of a suitable niche for the developing conceptus are indispensable [16,17]. IFNτ appears to be a key regulator of the fine-tuned maternal immunomodulation required for pregnancy establishment. IFNτ and all other type 1 interferons bind to the type 1 interferon receptor, which is formed by two subunits [18]. After interferon binding, the cell signal can be transmitted either via the classical pathway through the Janus‑activated kinase and signal transducer and activator of transcription (JAK‑STAT), or via non‑classical pathways, such as phosphoinositide 3-kinase (PI3K) [19]. Both classical and non‑classical pathways are essential for a successful and specific tissue response to different interferon stimuli, and this response is formed by classical and non‑classical interferon-stimulated genes (ISGs) [18].

In general, type 1 interferons, such as interferon α (IFNα), represent the first line of defense against viral infections. Unlike the others, IFNτ is not upregulated by viral infections [20], but has been described to have antibacterial activity in mice [21]. However, the most important quality of IFNτ is its key role in maternal recognition of pregnancy. This type 1 interferon inhibits pulsatile prostaglandin F-2 α release from the endometrium via the downregulation of estrogen receptor 1 and the oxytocin receptor, thereby preventing luteolysis of the corpus luteum and allowing pregnancy maintenance [22,23]. IFNτ further induces the expression of ISGs within the reproductive tract [24,25,26]. The respective proteins have immunomodulatory and metabolic functions and regulate growth and angiogenesis [27]. Minor alterations in the secretome composition have been reported to impair early pregnancy in cattle [28,29]. IFNτ is also known to have immunosuppressive functions. Recent studies indicate that day-seven embryos secrete IFNτ and trigger an anti-inflammatory response [30,31]. Exposure to paternal antigens occurs during embryo implantation. The immunomodulation of the maternal contact zone is required to avoid rejection, resorption, or expulsion of the conceptus [16,32]. Inflammatory processes can disturb the direct and indirect immunomodulatory actions of IFNτ in the maternal system. In the case of present viral infections, type 1 interferons other than IFNτ, such as IFNα, might disturb embryo–maternal crosstalk via competitive signaling.

**Figure 1:**
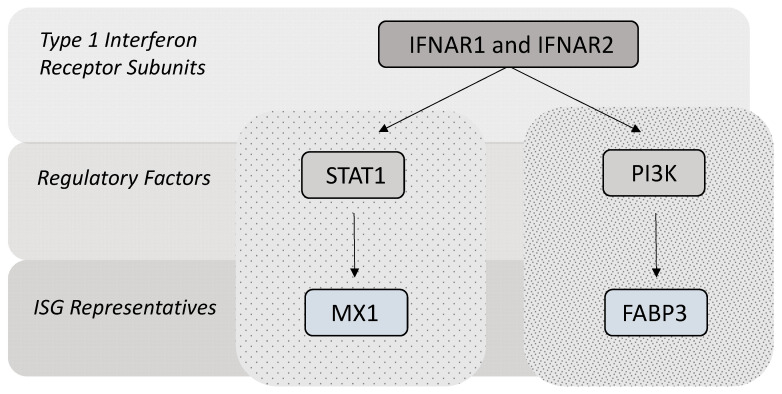
Schematic illustration of selected target gene representatives. Type 1 interferon receptor subunit 1 (IFNAR1); Type 1 interferon receptor subunit 2 (IFNAR2); signal transducer and activator of transcription 1 (STAT1); phosphoinositide 3-kinase (PI3K); interferon-stimulated gene (ISG); interferon-induced GTP-binding protein (MX1); fatty acid-binding protein 3 (FABP3).

In this study, six target genes were selected to exemplarily simulate both the classical and non‑classical pathways of type 1 interferon signaling (Figure 1). In this regard, the type 1 interferon receptor subunits 1 and 2 (IFNAR1 and IFNAR2) represent the top level of the signaling pathways for type 1 interferons. The intermediate level is represented by regulatory factors: the signal transducer and activator of transcription 1 (STAT1), exemplarily for classical type 1 interferon signaling, and phosphoinositide 3-kinase (PI3K), exemplarily for non-classical type 1 interferon signaling. At the basal level, interferon-induced GTP-binding protein (MX1, earlier myxomatose resistance protein 1) was selected, as it is one of the most prominent classical ISGs [33,34,35,36]. Fatty acid-binding protein 3 (FABP3) was chosen as a promising candidate that represents the non-classical pathway in bovine species in this study [37,38].

Although many researchers have contributed work to this field, it remains unknown why inflammatory conditions such as subclinical endometritis can lead to inadequate embryo–maternal crosstalk. Therefore, the aim of this project was to apply a highly defined endometrial explant model to achieve the following:

(a) Test the inducibility of type 1 interferon receptor subunits 1/2 (IFNAR1/2);
(b) Mimic a competitive situation between the embryonic signal IFNτ and other type 1 interferons (exemplarily IFNα) in the presence of progesterone (P4);
(c) Simulate exemplarily classical and non-classical type 1 interferon signaling pathways.

## 2. Materials and Methods

### 2.1. Donor Cows and Sample Criteria

Bovine uteri (n = 26) were collected at the local abattoir between May and October 2019. The breeds included Brown Swiss (n = 9), Simmental (n = 8), Holstein–Friesian (n = 6), Red Holstein (n = 1), and unknown breed (n = 2). The age of the donor cows ranged from 2.6 to 13.3 years. A pre-selection of the uteri was made right after evisceration. The selected organs were transferred to a separate room within the facility. A thorough adspectoral and palpatory examination for pathological changes, such as increased fluctuation, hemorrhage, mucosal lesions, and other anomalies, was then conducted. Only uteri that had not been contaminated during the process of slaughter were used for sample collection. All uteri that were selected for further sample collection were intact, symmetrical, and did not show any obvious pathological alterations. Additionally, the attached ovaries had at least one mature corpus luteum, indicating that the donor cow was in diestrus, and displayed no signs of pathological alterations.

### 2.2. Sample Collection

All instruments and materials were sterile and for each uterus, a new set of instruments and materials was applied. After cleaning and disinfecting the outer surface of the uterus with water and 70% ethanol, the uterine lumen ipsilateral to the mature corpus luteum was opened via a 2–3 cm longitudinal incision with a scalpel and forceps. The intercaruncular endometrial surface was first swabbed (UNI‑TER sterile swab with Amies medium, Vacutest Kima s.r.l., Arzergrande, Italy), and then cytobrushed (Celltip Cytobrush, Servoprax GmbH, Wesel, Germany) for further bacteriological and cytological examination. The cut was extended along the uterine body up to the horn tip and the tissue margins were pinned to an underlying styrofoam plate covered with tinfoil (Carl Roth, Karlsruhe, Germany). To ensure a continuative and reproducible size of endometrial explants, a disposable 5 mm biopsy punch (kai medical, Seki, Japan), precision forceps, and scissors were used, adapted from the method described by Borges et al. [39]. After gently pressing the biopsy punch through the intercaruncular endometrial and myometrial tissue, the explant was held in position with precision forceps and dissected with scissors (see Figure S1a). The obtained explants were placed in 60 mL sample vials (Thermo Fisher Scientific, Waltham, MA, USA) containing approximately 40 mL Dulbecco´s phosphate-buffered saline (PBS, Merck, Darmstadt, Germany) and stored on ice until further processing. From each donor cow, n = 28 (n = 728 in total) endometrial explants were obtained for the endometrial explant model. After sample collection had been completed, the uterus was separated from the ovaries, oviducts, and their mesentery, including the mesometrium, to measure the weight of the uterus. The time between the slaughter of the cow and the completion of sampling was <90 min.

### 2.3. Bacteriological Examination

Obtained bacteriological swabs of each sampled uterus were applied to non‑selective Columbia Sheep Blood Agar, *Streptococcus* selective Edwards Agar, and Enterobacteriaceae selective Violet Red Bile Agar, and incubated for 48 h at 37 °C. The agar plates were checked for bacterial growth at 24 h and 48 h of incubation.

### 2.4. Cytological Examination

Cytological examination of the endometrium was performed as previously described by Helfrich et al. [40]. In short, the collected cytobrush samples were smeared onto two glass slides and stained with Haema LT‑SYS Quick‑Stain (Labor + Technik Eberhard Lehmann GmbH, Berlin, Germany) in the laboratory. Under a microscope (Leitz, Stuttgart, Germany) using 100 × magnification and oil immersion, 300 nucleated cells were enumerated and the proportion of polymorphonuclear cells (PMN) was evaluated by two independent examiners [41]. A PMN proportion of ≥5% was classified as subclinical endometritis (SE) [42].

### 2.5. Leukocyte Esterase Test

After the cytological smear, the endometrial cytobrush was rinsed with 2 mL H_2_O and the fluid was probed with a Multistix^®^ 10 SG urine stick (Siemens Healthineers, Erlangen, Germany) to evaluate the leucocyte content after a 2 min incubation time, as previously described in low-volume lavage samples of the uterus [43,44].

### 2.6. Endometrial Explant Culture

In the laboratory, each step of the endometrial explant handling was performed in a class II biosafety cabinet (Biowizard Golden Line, Kojair, Vilppula, Finland). The uterus medium contained 99% Dulbecco´s modified Eagle´s medium/Nutrient Mixture f‑12 Ham (Merck) supplemented with 1% 10,000 µg/mL Penicillin–Streptomycin (Merck). Cellstar^®^ 24‑well culture plates (Greiner Bio‑One, Kremsmünster, Austria) containing 1 mL uterus medium were pre‑incubated (Heracell vios 160i, Thermo Fisher Scientific) at 37 °C, with 5% CO_2_ and 5.5% O_2_, for 1 h. Endometrial explants were placed in sterile glass Petri dishes with PBS. Remaining myometrial tissue was removed with precision forceps and a scalpel (see Figure S1b). After preparation, the explants were transferred into the pre‑incubated 24‑well culture plates, with one per well, and left to rest for 1 h at 4 °C. Meanwhile, IFNτ and IFNα (Cloud Clone Corp., Houston, TX, USA) were prepared with uterus medium in dilutions of 10, 100, and 300 ng/mL each. Furthermore, the IFNτ + IFNα combinations of 10 + 100, 100 + 10, and 100 + 100 ng/mL were prepared with uterus medium and 20 ng/mL P4 (pre‑diluted with uterus medium) was thawed. Only uterus medium (“control uterus medium”), uterus medium only with 100 ng/mL IFNτ (“control 100τ”), and uterus medium only with 20 ng/mL P4 (“control P4”) served as controls. After resting, the uterus medium was replaced by the prepared admixtures. The explants were challenged with IFNτ and/or IFNα in different concentrations in the presence of P4, according to the above-mentioned concentrations (see pipetting layout, Figure S2). The plate was incubated for 24 h at 37 °C, with 5% CO_2_ and 5.5% O_2_. These conditions had been evaluated as suitable in previous experiments [45]. After the completed incubation time, the endometrial explants were preserved in 0.5 mL cryogenic tubes (Sarstedt, Nürnbrecht, Germany) containing 450 µL RNA storage reagent RNAlater (Sigma‑Aldrich, St. Louis, MO, USA) at −80 °C. The supernatants were aspirated and preserved in 1.4 mL micronic tubes (Micronic, Lelystad, The Netherland) at −20 °C.

### 2.7. Determination of Tissue Viability

The tissue viability of the endometrial explants of each donor cow was determined with water‑soluble tetrazolium salt‑8 (WST‑8; Merck). WST‑8 is reduced to the orange–red formazan by dehydrogenases that are only present in viable cells. The amount of the color indicator formazan is thereby directly proportional to the metabolic cell activity of viable cells, and can be photometrically measured as the optical density (OD).

Therefore, four endometrial explants of each donor cow were handled identically for the challenge experiment until incubation. These explants were cultured in a separate 24-well culture plate with 1 mL uterus medium at 37 °C with 5% CO_2_ and 5.5% O_2_. Groups of two explants were treated at either 0 h or 21 h with WST‑8, and incubated for another 3 h. At the end of the respective incubation time (3 h/24 h), the supernatants of the incubated explants were aspirated and photometrically measured at a wavelength of 460 nm (CLARIOstar^®^, BMG LABTECH, Ortenberg, Germany) to determine the OD at 3 h and 24 h, as well as their percentage difference.

### 2.8. Gene Expression Analysis

For total RNA extraction, the explant duplicates of each animal were pooled in one innuSPEED Lysis Tube P (Analytic Jena AG, Jena, Germany) containing 450 µL lysis buffer for homogenization in a SpeedMill Plus device (Analytic Jena AG, Jena, Germany). The lysis buffer was part of the Bio&Sell RNA Mini Kit (Bio&SELL GmbH, Nürnberg, Germany) that was used according to the manufacturer´s instructions, with an additional DNase I digestion reaction using 50 µL DNase I reaction buffer and 5 µL DNase I (Bio&SELL GmbH). After RNA extraction, the approximately 80 µL eluate containing the purified RNA was subdivided: 2 µL was used for further RNA quality analysis, 10 µL for cDNA synthesis, and the residual volume for cryopreservation at −80 °C in a 0.2 mL reaction tube (Biozym Scientific GmbH, Hessisch Oldendorf, Germany). Additionally, 10 µL of one sample from each animal was used for minus‑reverse transcriptase (−RT) control synthesis. RNA quality analysis was performed with the automated Experion electrophoresis station with RNA StdSens Chips (Bio‑Rad Laboratories, Hercules, CA, USA), according to the manufacturer’s instructions, to estimate the level of RNA degradation using the RNA Quality Indicator (RQI). The RQI values range from 1 to 10, with the classifications “unacceptable quality”, (1–3), “possibly acceptable quality”, (4–6), and “acceptable quality”, (7–10).

Through reverse transcriptase, cDNA synthesis was performed directly after RNA extraction, according to the manufacturer’s 25 µL protocol, with 10 µL purified RNA and the following Promega reagents: 1 µL Oligo(dT)15 Primer, 0.625 µL RNasin^®^ Ribonuclease Inhibitor, 5 µL M‑MLV RT 5X Buffer, 1.25 µL dNTP Mix, 0.5 µL M‑MLV Reverse Transcriptase (Promega, Madison, WI, USA), and 6.625 µL PCR-grade water (Carl Roth). To synthesize the –RT control, reverse transcriptase synthesis was carried out in the absence of the reagent M‑MLV Reverse Transcriptase. The cDNA products were photometrically measured to determine their concentration and diluted to the end concentration of 200 ng/µL with PCR‑grade water (Carl Roth). The cDNA and –RT control products were then stored in 0.2 mL reaction tubes (Biozym Scientific GmbH) at −20 °C until further use.

A quantitative real‑time polymerase chain reaction (RT‑qPCR) was executed with the thermocycler qTOWER^3^ 84 G (Analytic Jena AG) and 384-well skirted PCR plates (FrameStar^®^, 4titude^®^ Limited, Wotton, Surrey, UK). The mastermix used consisted of 5 µL fluorescent dye (SensiFAST^TM^ SYBR No‑ROX Kit, Meridian Bioscience, Cincinnati, OH, USA), 0.4 µL forward/reverse primer, and 3.2 µL PCR-grade water. The applied primers for IFNAR1, IFNAR2, STAT1, PI3K, MX1, and FABP3 were designed with the Primer‑BLAST software (NCBI, Rockville Pike, MD, USA), purchased from Biomers (biomers.net GmbH, Ulm, Germany), and diluted with PCR-grade water from a starting concentration of 100 µM to an end concentration of 5 µM. Primer sequences are provided in Table 1.

Frozen cDNA samples, –RT controls, and primers were tempered for 5 min at 60 °C (on the BioShake iQ (Quantifoil Instruments GmbH, Jena, Germany)). The mastermix was prepared according to protocol in a class I biosafety cabinet (LTF Labortechnik, Wasserburg, Germany) and pipetted in 9 µL portions, adding 1 µL of each standard, no template control (NTC), sample cDNA, or –RT control. All measurements were performed in duplicates. Amplification was executed at 95 °C for 2 min, 40 cycles at 95 °C for 5 s, and 15 s at 60 °C. For each data point in every PCR run, a melting curve was conducted, starting from 60 °C and heating up in 0.5 °C steps until 95 °C. Analyzing the melting curve derivations, a single peak representing the calculated melting temperature of each target gene sequence was identified. The standard curve method for the absolute quantification of gene expression was carried out for each primer with the defined cDNA subclone concentrations of 10^2^, 10^3^, 10^4^, 10^5^, and 10^6^ copies/µL. The cDNA subclones were produced with the Promega cloning kit pGEM^®^‑T Easy Vector System (Promega) and sequenced for product verification at the Eurofins laboratory (Eurofins Genomics, Munich, Germany). PCR amplification efficiency was recorded for each PCR run and ranged from 90% to 109%, except for the target gene PI3K, with amplification efficiencies of 85%, and 87%.

### 2.9. Statistical Analysis and Graphical Illustration

Gene expression data were statistically analyzed with the R program for statistical computing (Version 4.0.3, 2020-10-10). A simple linear mixed effects model was used to study the IFNAR1, IFNAR2, STAT1, PI3K, MX1 and FABP3 parameters as fixed effects with a random effect of animal. The normality and homoscedasticity of residuals were assessed via visual residual-diagnostics. Due to the not-normally distributed and heteroskedastic residuals, data of all target genes in final models were log transformed (natural logarithm). Because of the exploratory approach of the study, the correction of the *p* values for multiple comparisons was not performed. Calculated differences with *p* < 0.1 were regarded as statistical tendencies. Differences with *p* < 0.05 were regarded as statistically significant. Data presented in the text are provided in the form of mean ± standard deviation (SD).

## 3. Results

### 3.1. Characterization of Sampled Uteri

The weights of the examined uteri were 630 g ± 149 g. Evaluation of the bacteriological swabs did not show any kind of bacterial growth. Cytological evaluation revealed that none of the uteri (n = 26) showed a PMN proportion ≥5%. The additional leukocyte esterase test did not indicate any leukocyte positive results. Therefore, all sampled uteri were classified as having bacteriologically and cytologically healthy endometria.

### 3.2. Successful Application of the Highly Defined Endometrial Explant Model

In this study, an endometrial explant model was successfully implemented in vitro. After WST‑8 incubation, the obtained OD of endometrial explant supernatants was 0.91 ± 0.22 (3 h) and 1.62 ± 0.36 (24 h). The measured OD proved the viability of the endometrial explants. The percent increase from the OD at 3 h to the OD at 24 h was 78.45%. This increase reflects the acclimatization to the new incubation environment. As additional quality control, RQI values were measured. RNA quality analysis showed a mean RQI value of 9.3 ± 0.4, and the RQI was ≥7.9 in all samples except for one, which had an RQI value of 6.8. For the absolute quantification of gene expression, cDNA subclones were generated and successfully applied to exemplarily demonstrate downstream type 1 interferon signaling via selected target genes.

**Figure 2:**
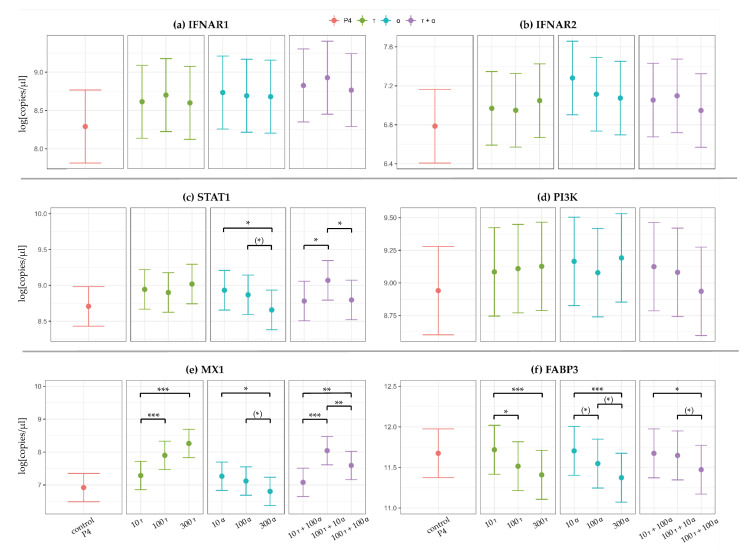
Illustration of the log-transformed gene expression of (**a**) type 1 interferon receptor subunit 1 (IFNAR1), (**b**) type 1 interferon receptor subunit 2 (IFNAR2), (**c**) signal transducer and activator of transcription 1 (STAT1), (**d**) phosphoinositide 3-kinase (PI3K), (**e**) interferon-induced GTP-binding protein (MX1), and (**f**) fatty acid-binding protein (FABP3) measured in bovine endometrial explants via a quantitative real-time polymerase chain reaction (RT-qPCR). X‑axis: explants were treated with 20 ng/mL progesterone (control P4, displayed in red) or with 20 ng/mL P4 and interferon τ (τ) and/or interferon α (α) at different concentrations (10, 100 and 300 ng/mL τ or α) and in different combinations (10τ + 100α, 100τ + 10α and 100τ + 100α). The Y‑axis represents log-transformed data of linear mixed effect models (natural logarithm). Significance codes: statistical tendencies *p* < 0.1 are marked with (*), statistical significances at *p* < 0.05, *p* < 0.01, and *p* < 0.001 are marked with *, **, and ***, respectively. Only significant differences within the three subsets τ (displayed in green), α (displayed in blue), and τ + α (displayed in purple) are marked with asterisks. Significant differences between the treatment group sets or between treatment groups and the control P4 are not marked in this graph, but can be found in Table S1a–f.

To mimic the physiological cycle stage diestrus during the challenge experiment, all endometrial explants were incubated in the presence of P4. Therefore, the expression of target genes was calculated and illustrated in relation to the control, “control P4”. The data concerning the controls “control uterus medium” and “control 100τ” served as internal validation. The log-transformed (natural logarithm) gene expression of target genes in relation to “control P4” is illustrated in Figure 2a–f. Each panel contains the following: (1) the “control P4” and the three treatment group sets; (2) IFNτ at three concentrations (10, 100, and 300 ng/mL); (3) IFNα at three concentrations (10, 100, and 300 ng/mL); and (4) the three combinations of IFNτ and IFNα (10 + 100, 100 + 10, and 100 + 100 ng/mL). Figure 2a–f includes statistically significant differences within these treatment group sets, indicated by asterisks. Significant differences between the treatment group sets and “control P4” are shown in Table S1a–f.

#### 3.2.1. Higher Expression of IFNAR1 and IFNAR2 After IFNα Challenge

Gene expression analysis confirmed the inducibility of IFNAR1/2 within the established endometrial explant model. In comparison to “control P4”, IFNAR1 was upregulated by trend in all explants challenged with IFNα (10, 100, and 300 ng/mL; *p* < 0.1) and in all explants challenged with combinations containing IFNα (*p* < 0.04). In comparison, only the gene expression after the challenge with 100 ng/mL IFNτ was upregulated (*p* = 0.066). Comparisons of the treatment group sets (Table S1a), as well as within treatment group sets (Figure 2a), did not show any significant differences concerning the gene expression of IFNAR1.

The induction of IFNAR2 was visible, as the gene expression was upregulated in all explants challenged with IFNα (*p* < 0.06) in comparison to “control P4”. Upregulation was most prominent with 10 ng/mL IFNα (*p* = 0.001). Comparisons of the treatment group sets showed a significantly higher gene expression in explants challenged with 10 ng/mL IFNα compared to 10 ng/mL IFNτ (*p* = 0.040), 100 ng/mL IFNτ (*p* = 0.029), and the combination of 100 ng/mL IFNτ + 100 ng/mL IFNα (*p* = 0.028) (Table S1b). There were no significant changes in IFNAR2 gene expression within treatment group sets (Figure 2b).

#### 3.2.2. IFNα Concentration-Dependent Gene Expression in STAT1

The target genes selected as representatives for regulatory factors concerning classical (exemplarily STAT1) and non-classical (exemplarily PI3K) interferon signaling were differentially regulated. Both target genes were upregulated in most treatment groups in comparison to “control P4” (Table S1c,d).

STAT1 gene expression was comparable between the treatment groups with IFNτ (10, 100, and 300 ng/mL; *p* > 0.1). The gene expression decreased at higher IFNα concentrations, with a significant difference between 10 and 300 ng/mL IFNα (*p* = 0.021) (Table S1c). When comparing the combinations of IFNτ and IFNα, the highest gene expression was detected in the treatment group with low IFNα (100 ng/mL IFNτ + 10 ng/mL IFNα versus 10 ng/mL IFNτ + 100 ng/mL IFNα (*p* = 0.016) and versus 100 ng/mL IFNτ + 100 ng/mL IFNα (*p* = 0.022); Figure 2c). Downregulation was most prominent in the 300 ng/mL IFNα group compared to the 100 ng/mL IFNτ + 10 ng/mL IFNα group (*p* < 0.001) (Table S1c). 

Concerning PI3K gene expression, only the combination of 100 ng/mL IFNτ + 100 ng/mL IFNα tended to be downregulated in comparison to 10 ng/mL IFNα (*p* = 0.096) and 300 ng/mL IFNα (*p* = 0.063) (Figure 2d, Table S1d). When comparing the combinations of IFNτ and IFNα, the lowest gene expression was detected in the treatment group with 100 ng/mL IFNα + 100 ng/mL IFNτ, but this was not significantly different in comparison to other combinations (*p* > 0.1) (Figure 2d).

#### 3.2.3. Type 1 Interferon-Specific, Concentration-Dependent Regulation of MX1 and FABP3

In comparison to “control P4”, MX1 gene expression was upregulated in most treatment groups, especially in those containing IFNτ (Table S1e). In contrast, FABP3 gene expression was similar or decreased in all treatment groups compared to “control P4” (Table S1f).

MX1 gene expression significantly increased along with rising IFNτ concentrations (10 versus 100 ng/mL (*p* < 0.001), 10 versus 300 ng/mL (*p* < 0.0001), and 100 versus 300 ng/mL (*p* = 0.026)). In comparison, rising IFNα concentrations led to a significant decrease in MX1 gene expression (10 versus 300 ng/mL (*p* = 0.005) and 100 versus 300 ng/mL (*p* = 0.052)) (Figure 2e). When comparing the combinations of IFNτ and IFNα, the highest gene expression was detected in the treatment group with low IFNα (100 ng/mL IFNτ + 10 ng/mL IFNα versus 10 ng/mL IFNτ + 100 ng/mL IFNα (*p* < 0.0001) and versus 100 ng/mL IFNτ + 100 ng/mL IFNα (*p* = 0.006); Figure 2e). Comparisons of the treatment group sets revealed the highest difference in gene expression between 300 ng/mL IFNτ and 300 ng/mL IFNα (*p* < 0.0001) (Table S1e).

In contrast to MX1, the gene expression of FABP3 significantly decreased along with rising IFNτ concentrations (10 versus 100 ng/mL (*p* = 0.029), and 10 versus 300 ng/mL (*p* < 0.001)). Identically to MX1, rising IFNα concentrations led to a decrease in FABP3 gene expression (10 versus 100 ng/mL (*p* = 0.089), 10 versus 300 ng/mL (*p* < 0.001), and 100 versus 300 ng/mL (*p* = 0.061)). When comparing the combinations of IFNτ and IFNα, the lowest gene expression was detected in the treatment group with 100 ng/mL IFNτ + 100 ng/mL IFNα (*p* < 0.06) (Figure 2f). 

Comparisons of the treatment group sets revealed the highest difference in gene expression between 10 ng/mL IFNτ and 300 ng/mL IFNα (*p* < 0.001) (Table S1f).

## 4. Discussion

The two adversaries IFNτ and IFNα were chosen to simulate the competitive situation between embryo–maternal communication and infection-associated signaling, which might impact subfertility in cattle. The applied concentrations of P4, IFNτ, and IFNα were chosen in accordance with those mentioned in the literature [46,47]. The targets IFNAR1/2, STAT1, PI3K, MX1, and FABP3 were selected and successfully applied to simulate exemplarily classical and non-classical type 1 interferon signaling pathways. The gene expression of these highly defined samples showed a significant upregulation of IFNAR1 and IFNAR2 after challenge with IFNτ and IFNα at differing concentrations and combinations, in comparison to “control P4”. Concentration-dependent upregulation of the classical ISG MX1 confirmed the validity of this model and its application for the simulation of the embryo–maternal contact zone.

In the literature, there is a limited number of studies focused on the establishment of endometrial explant models. Borges and coworkers established a model with intact endometrial tissue from bovine uteri [39], which focused on investigating the accumulation of differing interleukins in response to a challenge with heat-killed bacteria. The same explant-collecting technique was used by Mathew et al. to elucidate IFNτ-dependent and ‑independent effects on bovine endometrial gene expression [48]. Schäfer et al. tested human endometrial explants as suitable for analyzing the functional effects of chemicals outside the human body, and aspiration curettage was used to assess the endometrial tissue [49]. Bersinger and coworkers found that the cell morphology of human endometrial explants started to disrupt after 12 h in culture [50]. However, they were also able to show that this process was decelerated in the presence of P4. The advantage of explant models in contrast to cell culture models or chopped tissue is the maintenance of the integrity of the three-dimensional structure, which might reflect the tissue-specific reaction in a more accurate way. It was recently shown that endometrial explants from non-pregnant heifers collected on day 18 after insemination displayed a significantly higher gene expression of ISG-15 (classical ISG) when challenged with pregnancy-associated glycoproteins (PAG) after 24 h, in comparison to explants from non-pregnant heifers without PAG exposure [51]. This finding reflects a tissue-specific reaction towards a signal associated with early pregnancy. However, to the best of the authors’ knowledge, no bovine endometrial explant model has been previously applied to test the inducibility of type 1 interferon receptor subunits, and to mimic classical and non-classical type 1 interferon signaling pathways via exposure to IFNτ and IFNα at different concentrations and combinations.

In the present study, the gene expression results indicate a competitive situation between the two signals, IFNτ and IFNα, in the presence of P4. This effect was expected to be visible at the receptor level. Instead of that, this was detected downstream of the regulatory genes. The gene expression of IFNAR1 and IFNAR2 did not differ within the three treatment group sets. However, all treatment group sets with the combinations of IFNτ and IFNα exhibited significant differences downstream of the regulatory factor level, and along the classical type 1 interferon signaling pathway side (exemplarily STAT1 and MX1) and the non-classical type 1 interferon signaling pathway side (exemplarily PI3K and FABP3).

When comparing MX1 and STAT1 gene expressions in treatment group sets with combinations of IFNτ and IFNα, it was shown that higher IFNα concentrations led to decreased gene expression. It seems that with rising IFNα concentrations, entire successful IFNτ stimulation is prevented. These findings indicate that a competitive situation between IFNτ and IFNα can be simulated with this model on a regulatory factor level, and further downstream. Additional stressors, such as viral infections, might also lead to the parallel modulation of the type 1 interferon signaling cascade [10,52]. This might impede the discrimination of the systemic effects of IFNτ from those of other type 1 interferons. Bauersachs et al. [37] showed a wide overlap of endometrial gene expression in early pregnant heifers compared to heifers that underwent the intrauterine administration of IFNα. Furthermore, dairy cows are in a high lactation phase during pregnancy establishment. Metabolic challenges, in addition to immune‑endocrine changes, affect the intrauterine adaption capacity. This might affect important receptor expression and the complex intracellular downstream signaling of IFNτ in the endometrium, and can negatively affect embryo‑maternal communication. In relation to MX1 and STAT1, FABP3 and PI3K also showed similarities in their gene expression when comparing the treatment group sets with combinations of IFNτ and IFNα. Although the detected differences in FABP3 and PI3K are not as strong as in MX1 and STAT1, it can be noted that the treatment groups with the highest type 1 interferon concentrations have the lowest gene expression. This could also indicate a negative feedback response or dose effect due to excessive type 1 interferon signaling, or a counteraction of IFNα versus IFNτ. It might be possible that the competitive situation is also reflected by the gene expression data on the interferon receptor level, because both IFNAR1 and IFNAR2 gene expressions were higher after IFNα challenge, and the smallest amount of IFNα (10 ng/mL) led to the most prominent upregulation of IFNAR2. This could indicate regulative negative feedback or saturation mechanisms that might finetune the response to type 1 interferons at the receptor level. However, the evaluation of data in this direction is limited and highly interpretative, as only candidate gene expression data were considered.

It has been reported in multiple studies that ISGs are upregulated by IFNτ [33,37,53,54]. Our results are in line with these observations, as MX1 was significantly upregulated by IFNτ. Interestingly, MX1 was significantly downregulated by IFNα. In contrast, the non‑classical candidate FABP3 was downregulated by both type 1 interferons. These findings could reflect the necessity of a more adjusted IFNτ concentration approach, as only three concentrations were tested in this study. The physiological concentrations of IFNτ in vivo still need to be determined. This is a major task, because IFNτ levels are difficult to measure, as reviewed by Hansen et al. [55]. Concentrations of IFNτ applied in other in vitro studies ranged from 0.025 ng/mL [56] to 10,000 ng/mL [57]. In addition, the definition and described characteristics of non-classical ISGs vary between studies [58,59,60]. Predominantly non-classical ISGs are described to be induced via P4, and exhibit biological functions associated with pregnancy establishment [55,61,62]. In the present study, FABP3 was selected as a promising candidate to represent non-classical ISGs, because it had previously been described as P4-dependent and functionally involved in the modulation of cell growth and proliferation [58]. It was also promising because Bauersachs et al. [37] described FABP3 as being strongly upregulated on day 15 and 18 of pregnancy, but not after a challenge with IFNα. This group even described a significant decrease in FABP3 between day 13 and 19 in the estrus cycle in the same study. Unexpectedly, FABP3 was downregulated by differing concentrations of IFNτ and IFNα in our study, which has not been described before. Whether FABP3 is a suitable candidate for non-classical ISGs needs to be investigated. However, the model applied in this study could allow the evaluation of typical characteristics and the identification of the differences between classical and non-classical ISGs in future approaches.

Taken together, the present data generated with our highly defined endometrial explant model, used to simulate the embryo–maternal contact zone, indicate a competitive situation between IFNτ and IFNα. Of course, the interpretation of functionality is limited. In vitro models only reflect a snapshot of a far more complex situation in vivo. The implementation of data on the protein level would be a logical and desirable next step, but was outside of the feasible scope of this study. Furthermore, type 1 interferon signaling pathways are very complex, and no signaling pathway alone is considered sufficient to generate a complete biological response. Further studies with holistic techniques, such as transcriptomics and proteomics, are required to determine why different signal-transmitting possibilities are chosen over others. The possible applications of this model include simulations of inflammatory conditions, such as endometritis.

## 5. Conclusions

In conclusion, a highly defined endometrial explant model was successfully implemented in vitro and applied to test the inducibility of type 1 interferon receptor subunits (IFNAR1/2). Exemplarily, classical and non-classical type 1 interferon pathways were simulated via selected target genes. Further in vitro studies with this model might mimic and explore the embryo–maternal contact zone in more detail, to answer research questions in this area. This might lead to new diagnostic and therapeutic strategies for tackling subfertility in dairy cows without the need for animal experiments.

## Figures and Tables

**Table 1:** Oligonucleotide primer sequences used in the quantitative real-time polymerase chain reaction (RT‑qPCR) with the accession number and fragment size in base pairs (bp).

Gene	Accession Number	Forward (F) and Reverse (R) primer (5´‑3’)	Fragment Size
IFNAR1 ^1^	NM_174552.2	F: ACAGGCGGAATAAAGGGAGC R: AAGGCAGGTCCAATGACAGG	683 bp
IFNAR2 ^2^	NM_174553.2	F: GTGGGTAAACACGACGGACA R: CTCGTCTGGGTCGAAAGAGG	419 bp
STAT1 ^3^	NM_001077900.1	F: CGGTCCCAAAATGGAGGTGA R: ACATGCCACTCTTCTGTGTTCA	308 bp
PI3K ^4^	NM_174575.1	F: CTGAAGCAGACAGTGAGCAA R: CCAAGGAGGCGGTATCACAA	207 bp
MX1 ^5^	NM_173940.2	F: TTGGGAATGAAGACGAGTGG R: CCTCTGTGGTAGCGATGTCC	342 bp
FABP3 ^6^	NM_174313.2	F: TCGGTGTCGGTTTTGCTACC R: TCAACCATCTCCCGCACAAG	262 bp

^1^ Type 1 interferon receptor subunit 1; ^2^ type 1 interferon receptor subunit 2; ^3^ signal transducer and activator of transcription 1; ^4^ phosphoinositide 3-kinase; ^5^ interferon-induced GTP-binding protein; ^6^ fatty acid-binding protein.

## Data Availability

Data are contained within the article or supplementary material. Raw data are available on request from the corresponding author.

## Supplementary Materials

The following are available online at https://www.mdpi.com/2076-2615/11/2/262/s1: Figure S1 (a) and (b), graphical illustration of sampling procedure; Figure S2, graphical illustration of pipetting layout for the 24‑well culture plate; Table S1, (**a**) IFNAR1, (**b**) IFNAR2, (**c**) STAT1, (**d**) PI3K, (**e**) MX1, and (**f**) FABP3: *p* values of gene expression comparisons of treatment groups and the control (“control P4”).
