# Supplementary material for: Concentration-Dependent Type 1 Interferon-Induced Regulation of MX1 and FABP3 in Bovine Endometrial Explants
Source: Animals (Basel). 2021 Jan 21;11(2):262. doi: 10.3390/ani11020262 (PMC7912598; doi:10.3390/ani11020262)
Supplement: Supplementary file 1 [file animals-11-00262-s001.pdf]

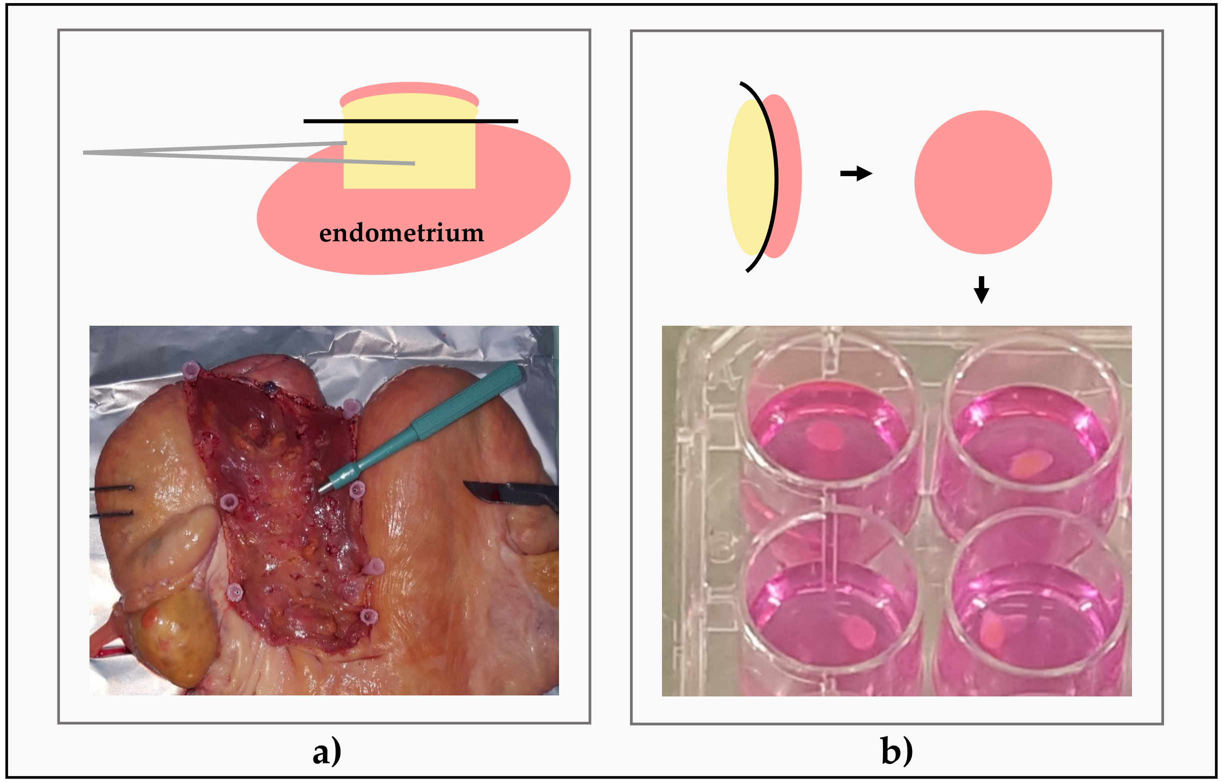

Figure S1. Graphical illustration of sampling procedure. Endometrial tissue displayed in pink, myometrial tissue displayed in yellow, dissections displayed as black lines, precision forceps displayed in gray. (**a**) Obtaining endometrial explants with a 5 mm biopsy punch, held in position with precision forceps and dissected from the myometrium (photo below, draft above). (**b**) Separating residual myometrial tissue from endometrial explants (draft above) and transfer to the 24-well culture plate (photo below).

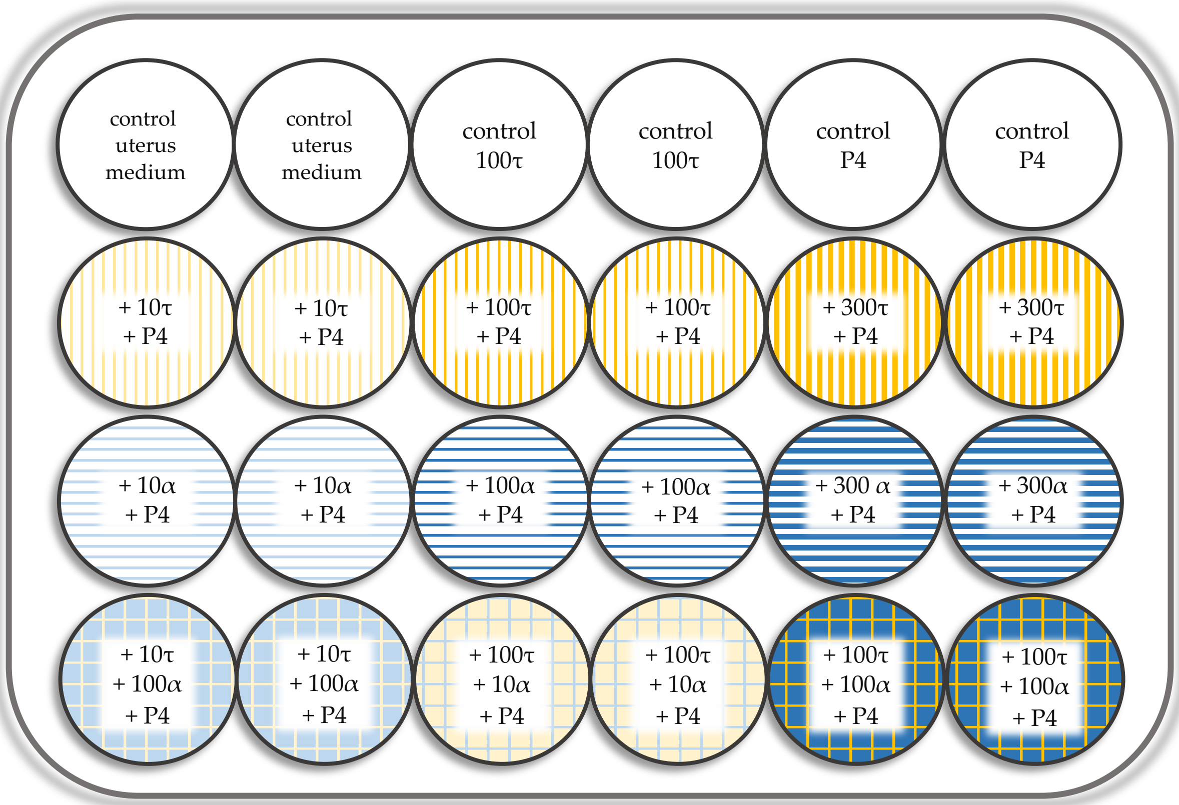

Figure S2. Graphical illustration of pipetting layout for the 24-well culture plate. Each well contains 1 mL uterus medium and respective admixtures interferon τ (τ), interferon α (α) in differing concentrations and combinations [ng/mL], additionally 20 ng/mL progesterone (P4) were added to indicated wells.

Table S1a). IFNAR1[1]: *p* values of gene expression comparisons between treatment groups and "control P4"

Treatment Group [ng / ml][2]	10τ	100τ	300τ	10α	100α	300α	10τ + 100α	100τ + 10α	100τ + 100α	control P4
**10τ**		n.s.	n.s.	n.s.	n.s.	n.s.	n.s.	n.s.	n.s.	n.s.
**100τ**	n.s.		n.s.	n.s.	n.s.	n.s.	n.s.	n.s.	n.s.	0.066
**300τ**	n.s.	n.s.		n.s.	n.s.	n.s.	n.s.	n.s.	n.s.	n.s.
**10α**	n.s.	n.s.	n.s.		n.s.	n.s.	n.s.	n.s.	n.s.	0.047
**100α**	n.s.	n.s.	n.s.	n.s.		n.s.	n.s.	n.s.	n.s.	0.072
**300α**	n.s.	n.s.	n.s.	n.s.	n.s.		n.s.	n.s.	n.s.	0.081
**10τ + 100α**	n.s.	n.s.	n.s.	n.s.	n.s.	n.s.		n.s.	n.s.	0.017
**100τ + 10α**	n.s.	n.s.	n.s.	n.s.	n.s.	n.s.	n.s.		n.s.	0.005
**100τ + 100α**	n.s.	n.s.	n.s.	n.s.	n.s.	n.s.	n.s.	n.s.		0.034
**control P4**	n.s.	0.066	n.s.	0.047	0.072	0.081	0.017	0.005	0.034	

[1] Type 1 interferon receptor subunit 1 (IFNAR1); [2] Log transformed gene expression of target genes was measured in bovine endometrial explants via real-time polymerase chain reaction (RT-qPCR) for every treatment group. Explants were treated with 20 ng/mL progesterone ("control P4") or with 20 ng/mL P4 and interferon τ (τ) and/or interferon α (α) at different concentrations (10, 100 and 300 ng/mL τ or α), and combinations (10τ + 100α, 100τ + 10α and 100τ + 100α). Comparisons of treatment groups were calculated with a linear mixed effect models. Significance codes: *p* values > 0.1 were regarded as not significant (n.s.), *p* values < 0.1 as statistical tendencies and *p* < 0.05 as statistically significant.

Table S1b). IFNAR2[3]: *p* values of gene expression comparisons between treatment groups and "control P4"

Treatment Group [ng / ml][2]	10τ	100τ	300τ	10α	100α	300α	10τ + 100α	100τ + 10α	100τ + 100α	control P4
**10τ**		n.s.	n.s.	0.040	n.s.	n.s.	n.s.	n.s.	n.s.	n.s.
**100τ**	n.s.		n.s.	0.029	n.s.	n.s.	n.s.	n.s.	n.s.	n.s.
**300τ**	n.s.	n.s.		n.s.	n.s.	n.s.	n.s.	n.s.	n.s.	0.083
**10α**	0.040	0.029	n.s.		n.s.	n.s.	n.s.	n.s.	0.028	0.001
**100α**	n.s.	n.s.	n.s.	n.s.		n.s.	n.s.	n.s.	n.s.	0.030
**300α**	n.s.	n.s.	n.s.	n.s.	n.s.		n.s.	n.s.	n.s.	0.057
**10τ + 100α**	n.s.	n.s.	n.s.	n.s.	n.s.	n.s.		n.s.	n.s.	0.077
**100τ + 10α**	n.s.	n.s.	n.s.	n.s.	n.s.	n.s.	n.s.		n.s.	0.040
**100τ + 100α**	n.s.	n.s.	n.s.	0.028	n.s.	n.s.	n.s.	n.s.		n.s.
**control P4**	n.s.	n.s.	0.083	0.001	0.030	0.057	0.077	0.040	n.s.	

[3] Type 1 interferon receptor subunit 2 (IFNAR2)

Table S1c). STAT1[4]: *p* values of gene expression comparisons between treatment groups and "control P4"

Treatment Group [ng / ml][2]	10τ	100τ	300τ	10α	100α	300α	10τ + 100α	100τ + 10α	100τ + 100α	control P4
**10τ**		n.s.	n.s.	n.s.	n.s.	0.016	n.s.	n.s.	n.s.	0.048
**100τ**	n.s.		n.s.	n.s.	n.s.	0.042	n.s.	n.s.	n.s.	n.s.
**300τ**	n.s.	n.s.		n.s.	n.s.	0.003	0.046	n.s.	0.062	0.009
**10α**	n.s.	n.s.	n.s.		n.s.	0.021	n.s.	n.s.	n.s.	0.060
**100α**	n.s.	n.s.	n.s.	n.s.		0.077	n.s.	0.089	n.s.	n.s.
**300α**	0.016	0.042	0.003	0.021	0.077		n.s.	< 0.001	n.s.	n.s.
**10τ + 100α**	n.s.	n.s.	0.046	n.s.	n.s.	n.s.		0.016	n.s.	n.s.
**100τ + 10α**	n.s.	n.s.	n.s.	n.s.	0.089	< 0.001	0.016		0.022	0.003
**100τ + 100α**	n.s.	n.s.	0.062	n.s.	n.s.	n.s.	n.s.	0.022		n.s.
**control P4**	0.048	n.s.	0.009	0.060	n.s.	n.s.	n.s.	0.003	n.s.	

[4] Signal transducer and activator of transcription (STAT1)

Table S1d). PI3K[5]: *p* values of gene expression comparisons between treatment groups and "control P4"

Treatment Group [ng / ml][2]	10τ	100τ	300τ	10α	100α	300α	10τ + 100α	100τ + 10α	100τ + 100α	control P4
**10τ**		n.s.	n.s.	n.s.	n.s.	n.s.	n.s.	n.s.	n.s.	n.s.
**100τ**	n.s.		n.s.	n.s.	n.s.	n.s.	n.s.	n.s.	n.s.	n.s.
**300τ**	n.s.	n.s.		n.s.	n.s.	n.s.	n.s.	n.s.	n.s.	n.s.
**10α**	n.s.	n.s.	n.s.		n.s.	n.s.	n.s.	n.s.	0.096	n.s.
**100α**	n.s.	n.s.	n.s.	n.s.		n.s.	n.s.	n.s.	n.s.	n.s.
**300α**	n.s.	n.s.	n.s.	n.s.	n.s.		n.s.	n.s.	0.063	0.069
**10τ + 100α**	n.s.	n.s.	n.s.	n.s.	n.s.	n.s.		n.s.	n.s.	n.s.
**100τ + 10α**	n.s.	n.s.	n.s.	n.s.	n.s.	n.s.	n.s.		n.s.	n.s.
**100τ + 100α**	n.s.	n.s.	n.s.	0.096	n.s.	0.063	n.s.	n.s.		n.s.
**control P4**	n.s.	n.s.	n.s.	n.s.	n.s.	0.069	n.s.	n.s.	n.s.	

[4] Phosphoinositide 3-kinase (PI3K)

Table S1e). MX1[6]: *p* values of gene expression comparisons between treatment groups and "control P4"

Treatment Group [ng / ml][2]	10τ	100τ	300τ	10α	100α	300α	10τ + 100α	100τ + 10α	100τ + 100α	control P4
**10τ**		< 0.001	< 0.0001	n.s.	n.s.	0.003	n.s.	< 0.0001	0.059	0.024
**100τ**	< 0.001		0.026	0.0001	< 0.0001	< 0.0001	< 0.0001	n.s.	0.059	< 0.0001
**300τ**	< 0.0001	0.026		< 0.0001	< 0.0001	< 0.0001	< 0.0001	n.s.	< 0.0001	< 0.0001
**10α**	n.s.	0.0001	< 0.0001		n.s.	0.005	n.s.	< 0.0001	0.043	0.034
**100α**	n.s.	< 0.0001	< 0.0001	n.s.		0.052	n.s.	< 0.0001	0.004	n.s.
**300α**	0.003	< 0.0001	< 0.0001	0.005	0.052		0.091	< 0.0001	< 0.0001	n.s.
**10τ + 100α**	n.s.	< 0.0001	< 0.0001	n.s.	n.s.	0.091		< 0.0001	0.002	n.s.
**100τ + 10α**	< 0.0001	n.s.	n.s.	< 0.0001	< 0.0001	< 0.0001	< 0.0001		0.006	< 0.0001
**100τ + 100α**	0.059	0.059	< 0.0001	0.043	0.004	< 0.0001	0.002	0.006		< 0.0001
**control P4**	0.024	< 0.0001	< 0.0001	0.034	n.s.	n.s.	n.s.	< 0.0001	< 0.0001	

[6] Interferon-induced GTP-binding protein (MX1)

Table S1f). FABP3[7]: *p* values of gene expression comparisons between treatment groups and "control P4"

Treatment Group [ng / ml][2]	10τ	100τ	300τ	10α	100α	300α	10τ + 100α	100τ + 10α	100τ + 100α	control P4
**10τ**		0.029	< 0.001	n.s.	0.065	< 0.001	n.s.	n.s.	0.008	n.s.
**100τ**	0.029		n.s.	0.041	n.s.	n.s.	0.088	n.s.	n.s.	0.086
**300τ**	< 0.001	n.s.		0.002	n.s.	n.s.	0.004	0.01	n.s.	0.004
**10α**	n.s.	0.041	0.002		0.089	< 0.001	n.s.	n.s.	0.012	n.s.
**100α**	0.065	n.s.	n.s.	0.089		0.061	n.s.	n.s.	n.s.	n.s.
**300α**	< 0.001	n.s.	n.s.	< 0.001	0.061		0.001	0.003	n.s.	0.001
**10τ + 100α**	n.s.	0.088	0.004	n.s.	n.s.	0.001		n.s.	0.030	n.s.
**100τ + 10α**	n.s.	n.s.	0.01	n.s.	n.s.	0.003	n.s.		0.058	n.s.
**100τ + 100α**	0.008	n.s.	n.s.	0.012	n.s.	n.s.	0.03	0.058		0.029
**control P4**	n.s.	0.086	0.004	n.s.	n.s.	0.001	n.s.	n.s.	0.029	

[7] Fatty acid-binding protein 3 (FABP3)
